# The Prognostic Value of Decreased KLF4 in Digestive System Cancers: A Meta-Analysis from 17 Studies

**DOI:** 10.1155/2017/3064246

**Published:** 2017-09-14

**Authors:** Jianpei Hu, Huipu Li, Chunyu Wu, Xueying Zhao, Chaodong Liu

**Affiliations:** ^1^Department of Urology, The First Affiliated Hospital of Chongqing Medical University, Chongqing, China; ^2^Department of Gastroenterology, The First Affiliated Hospital of Chongqing Medical University, Chongqing, China

## Abstract

**Background:**

The prognostic value of loss of Krüppel-like factor 4 (KLF4) expression in digestive system cancers has not reached a consensus. This study aimed for a comprehensive investigation of the internal associations between KLF4 expression loss and prognostic implications in patients with digestive system cancers.

**Methods:**

We searched for all relevant literatures in the electronic databases until February 1, 2017. The degree of association between KLF4 and prognosis was evaluated by pooled hazard ratios (HRs) as well as relevant 95% confidence intervals (95% CIs).

**Results:**

Seventeen eligible studies with 2118 patients revealed that loss of KLF4 expression was connected with poor prognosis, with the pooled HRs of 1.61 (95% CI: 1.17–2.20, *P* = 0.003) for the overall survival (OS) and 1.99 (95% CI: 1.12–3.52, *P* = 0.001) for the disease-free survival (DFS)/recurrence-free survival (RFS)/metastasis-free survival (MFS). Additionally, loss of KLF4 expression was also related to a worse disease-special survival (DSS) yielding a pooled HR of 1.73 (95% CI: 1.08–2.77, *P* = 0.022).

**Conclusion:**

Our findings suggest that loss of KLF4 expression is correlated with a bad outcome in most digestive system cancers, apart from esophagus squamous cell carcinoma (ESCC).

## 1. Introduction

Digestive system cancers generally refer to these cancers that arise from the esophagus, stomach, liver, gallbladder, biliary tract, colon, rectum, and anus, and all of them are common types of carcinomas around the world. Of note, colorectal, gastric, and liver cancer are the leading causes of cancer-related deaths which therefore confer a heavy burden on the society worldwide [1]. In the United States, there are approximately 310,440 new cases diagnosed with digestive system cancers with an estimated 157,700 deaths in 2017 [2]. On the one hand, despite a vast number of progresses have been made for the etiology, diagnosis and therapy of digestive system malignancies, the prognoses of these patients are still poor and unsatisfied; on the other hand, the advent of the molecular-targeted therapy era provides new choices of cancer therapy with a promising prospect [3, 4]. Hence, much more efforts should be made by researchers to identify those ideal molecular markers that represent both therapeutic value and predictive value for prognosis, then contributing to risk stratification and optimal choice of treatment for patients.

Krüppel-like factor 4 (KLF4) can also be referred to as gut-enriched KLF (GKLF) or epithelial zinc finger protein (EZF) which mainly expresses in epithelial tissues of the mammals, including the intestine, skin, thymus, and lung. As a complicated transcription factor, KLF4 contains a highly conserved C-terminal DNA-binding domain with three zinc fingers. In physiological condition, upon binding to the specific sequences, including CACCC boxes and GC boxes, KLF4 can exert multiple functions through regulating many cellular processes, such as cell proliferation, development, apoptosis, and homeostasis [5, 6]. Furthermore, in the context of most malignancies, KLF4 is necessary for the suppression of tumorigenesis and progression, basing on its inhibition of epithelial-mesenchymal transition (EMT), cell proliferation, and migration [7–10]. However, it has also been reported that KLF4 may be an oncogene in a few types of cancers, such as breast cancer and skin squamous cell carcinoma [11, 12], suggesting that KLF4, similar to transforming growth factor-*β* and Notch, may have opposing roles in tumorigenesis and progression in a context-dependent manner [13, 14].

In the context of digestive system cancers, the vast majority of studies have revealed that KLF4 is decreased or absent with a bad clinical outcome, including esophagus squamous cell carcinoma (ESCC) [15, 16], gastric cancer (GC) [17–19], pancreatic ductal adenocarcinoma (PDAC) [20, 21], hepatocellular cancer (HCC) [22–24], and colorectal cancer (CRC) [25–27]. However, the dependability of KLF4 serving as a prognostic biomarker has not been coming to an agreement in different cancers for the insignificant even opposite results [28–31]. Hence, the prognostic role of KLF4 in patients with digestive system cancers remains disputed. It is therefore unknown that the differences in these studies are most caused by their small sample size or inherent heterogeneity. On account of the limits of a single study, it is necessary to evaluate the reported studies using a comprehensive meta-analysis.

In this study, the goal is to determine the prognostic value of loss of KLF4 expression among digestive system cancers via gathering global relevant literatures to perform a systematic analysis.

## 2. Materials and Methods

### 2.1. Search Strategy

A thorough search was carried out for all relevant literatures that evaluated the prognostic value of KLF4 in different digestive system cancers until February 1, 2017 among the following electronic databases: Pubmed, ISI Web of Science and Embase. Search terms represented as follows: (KLF4 OR Krüppel-like factor 4 OR Gut-enriched KLF OR GKLF OR ZEF OR Epithelial Zinc Finger Protein) AND (cancer OR tumor OR neoplasm OR carcinoma) AND (Prognosis OR prognostic OR survival OR outcome). The Cochrane Library was also reviewed for related papers. In addition, the citation lists of identified articles were manually reviewed to complete the search. Two authors (Hu and Li) independently performed this procedure. Any disagreement was resolved by mutual discussion.

### 2.2. Selection Criteria

In this meta-analysis, the eligibility of candidate studies was determined based on the following criteria: (i) studied the patients with digestive system cancers; (ii) measured KLF4 expression using either semiquantitative immunohistochemistry (IHC) or quantitative reverse transcription PCR (RT-PCR); and (iii) evaluated the correlation between KLF4 expression and prognosis. Articles were not taken into account when the following criteria were met: (i) duplicated or overlapped studies; (ii) reviews, case reports, comments, or conference abstracts; and (iii) absence of key information for further quantification calculation. Two individuals (Zhao and Wu) separately carried out all evaluations and any discrepancy was resolved by consensus.

### 2.3. Quality Assessment

To accomplish the process of quality assessment, each eligible article was scored in the light of the Newcastle-Ottawa scale (NOS) [32] because all of them were observational studies. The cohorts of included studies were scored in terms of selection, comparability, and outcome and yielded a total score up to 9 points. Generally, NOS scores ≥ 6 was considered to indicate high-quality studies in methodology [33]. After independent assessment by two authors (Hu and Zhao), a joint decision was made in the case of any discrepancy.

### 2.4. Data Extraction and Conversion

Data retrieved from the reports included the following elements: author, publication year, origin of population, tumor type, follow-up time, sample size, KLF4 measurement method, cut-off value, the HRs, and 95% CIs of KLF4 for OS, DFS, MFS, RFS, and DSS. The original survival data were obtained from the text, tables or Kaplan-Meier curves for both comparative groups. Engauge Digitizer 4.1 (downloaded from http://markummitchell.github.io/engauge-digitizer) helped us to digitize and to extract survival information from the Kaplan-Meier curves using the method established by Tierney et al. [34]. Two individuals (Hu and Li) independently undertook this process to warrant the precision and a joint decision was made on the occasion of disparity.

### 2.5. Statistical Analysis

The HRs in combination with the corresponding 95% CIs of identified studies were combined to estimate the overall effective value following *Tierney*'s method [34]. Cochran's Q test and Higgin's *I*^2^ statistics were simultaneously adopted for the test of heterogeneity of combined HRs [35]. A random effects model was adopted to aggregate the pooled HR when significant heterogeneity existed (*P* < 0.10 and/or *I*^2^ > 50%); on the contrary, a fixed effects model was employed (*P* > 0.10 and/or *I*^2^ < 50%). The impact of decreased KLF4 expression on the prognosis was measured by the combined HRs and its corresponding 95% confidence intervals extracted from each included article. Indirect HRs with related 95% CIs were obtained via the method established by Tierney. Generally, a pooled HR of >1 was assumed to indicate a significant association with poor prognosis and was interpreted as statistically significant when its 95% CI did not cross 1. Both Begg's test and Egger's test were done to judge the probability of publication bias. Sensitivity analysis, aiming for evaluation of the stability of results, was put into effect by removing each individual study at every turn. Two-sided *P* < 0.05 possessed statistical significance. All analyses used in the meta-analysis were performed by way of STATA version 13.0 (Stata Corporation, College Station, TX).

## 3. Results

**Figure 1:**
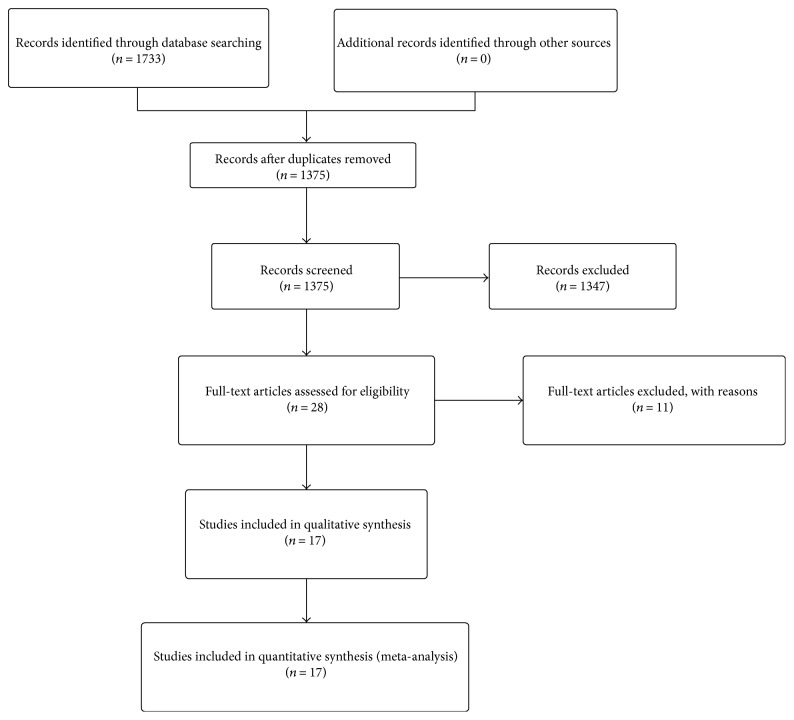
Selection flow chart of the meta-analysis.

According to the pre-established inclusion criteria, most of the preliminarily included entries were eliminated on account of duplicated data, inappropriate article type, or inadequate original information. Eventually, a total of 17 observational studies consisting of 2188 cases were retained for subsequent pooling calculation. The selection procedure of all eligible studies in our meta-analysis was summarized concisely in Figure 1.

### 3.1. Demographic Characteristics of Included Studies

As for the source regions of included studies, the majority were carried out in China (*n* = 12), followed by the USA (*n* = 2) and other sporadic nations. None of the eligible entries scored less than six by the Newcastle-Ottawa scale, revealing a high methodological quality across all studies. Studies concerning colorectal cancer occupied the largest proportion of cancer type among all primary literatures (*n* = 5), followed by HCC (*n* = 4), GC (*n* = 3), ESCC (*n* = 3), and PDAC (*n* = 2). The sample size of identified articles ranged from 22 to 365, with a mean of 128 patients. A total of 15 studies described the correlation of overall survival and KLF4 deficiency, while 9 trials reported a relationship between other survival parameters and KLF4 absence. The rest of the detailed features were recorded and summarized in Table 1.

### 3.2. Meta-Analysis

**Figure 2:**
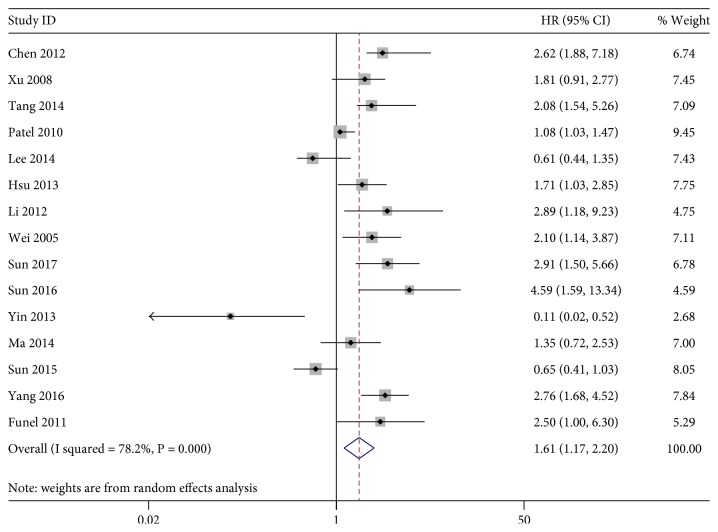
Forest plot of studies evaluating HRs of loss of KLF4 expression for OS.

**Figure 3:**
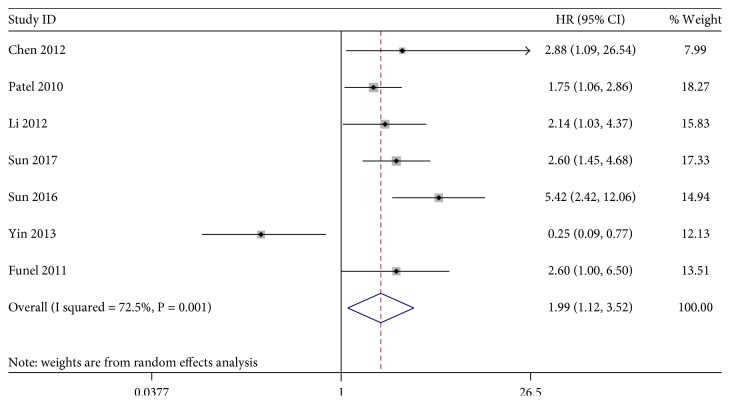
Forest plot of studies evaluating HRs of loss of KLF4 expression for DFS/RFS/MFS.

**Figure 4:**
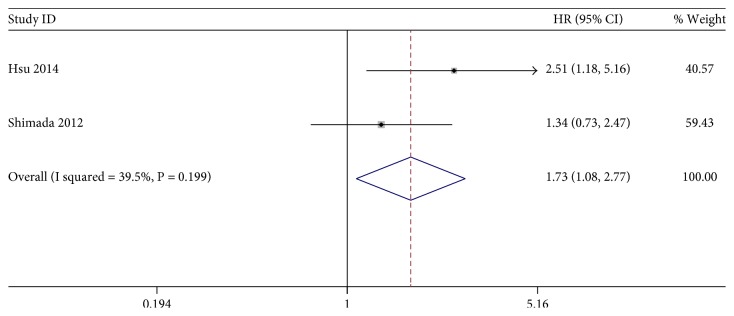
Forest plot of studies evaluating HRs of loss of KLF4 expression for DSS.

The association between KLF4 expression loss and digestive system cancer prognosis was illustrated in Figures 2, 3, and 4. Overall, loss of KLF4 expression had a bad outcome in those patients, with the pooled HRs of 1.61 (95% CI: 1.17–2.20, *P* = 0.003) for OS via a random model because of the significant heterogeneity (*I*^2^ = 78.2%, *P* = 0.001). Additionally, negative KLF4 expression was also correlated with a poorer disease-free survival (DFS)/recurrence-free survival (RFS)/metastasis-free survival (MFS), with the pooled HR of 1.99 (95% CI: 1.12–3.52, *P* = 0.019) calculated by a random model because of the presence of profound heterogeneity (*I*^2^ = 72.5%, *P* = 0.001). At last, KLF4 was connected with disease-special survival (DSS), with the pooled HR of 1.73 (95% CI: 1.08–2.77, *P* = 0.022) through a fixed effects model for insignificant heterogeneity (*I*^2^ = 39.5%, *P* = 0.199).

To explore the sources of heterogeneity, subgroup analyses for OS and DFS/RFS/MFS were conducted by the ethnicity, measurement method, and cancer types. The main results of this subgroup analyses for the prognostic role of KLF4 deficiency in digestive system cancers were shown in Table 2. In the ethnicity subgroup analyses, considerable heterogeneity was observed in both groups for OS and DFS/RFS/MFS; the results showed that KLF4 expression loss reduced significantly the OS (HR = 1.54, 95% CI: 1.28–1.84, *P* = 0.001) and DFS/RFS/MFS (HR = 1.91, 95% CI: 1.23–1.96, *P* = 0.001) in Asian cancer patients as well as the OS in Caucasian patients (HR = 1.17, 95% CI: 1.00–1.38, *P* = 0.07), but not the DFS/RFS/MFS in Caucasian ones (HR = 0.59, 95% CI: 0.36–0.94, *P* = 0.004).

In the subgroup analyses by the measurement method, the results revealed that decreased expression of KLF4, in the IHC group, produced a poorer prognosis for OS (HR = 1.38, 95% CI: 1.21–1.57, *P* = 0.002) and DFS/RFS/MFS (HR = 2.43, 95% CI: 1.82–3.25, *P* = 0.001), but not in RT-PCR ones for OS (HR = 0.93, 95% CI: 0.62–1.39, *P* = 0.081) and DFS/RFS/MFS (HR = 0.25, 95% CI: 0.09–0.73, *P* = 0.001). However, we also found that there was a significant heterogeneity for OS as well as DFS/RFS/MFS in those subgroups.

In the stratified analyses according to cancer type, expression loss of KLF4 yielded a poorer OS in CRC (HR = 1.17, 95% CI: 1.01–1.37), GC (HR = 1.97, 95% CI: 1.36–2.83, *P* = 0.015), HCC (HR = 2.30, 95% CI: 1.35–3.92, *P* = 0.001), and PDAC (HR = 2.70, 95% CI: 1.75–4.17) and a worse DFS/RFS/MFS in CRC (HR = 1.83, 95% CI: 1.14–2.94), HCC (HR = 2.20, 95% CI: 1.43–3.39), GC (HR = 2.14, 95% CI: 1.04–4.41), and PDAC (HR = 2.60, 95% CI: 1.02–6.63), but not statistically significant in ESCC for OS (HR = 0.84, 95% CI: 0.58–1.22).

### 3.3. Publication Bias and Sensitivity Analysis

**Figure 5:**
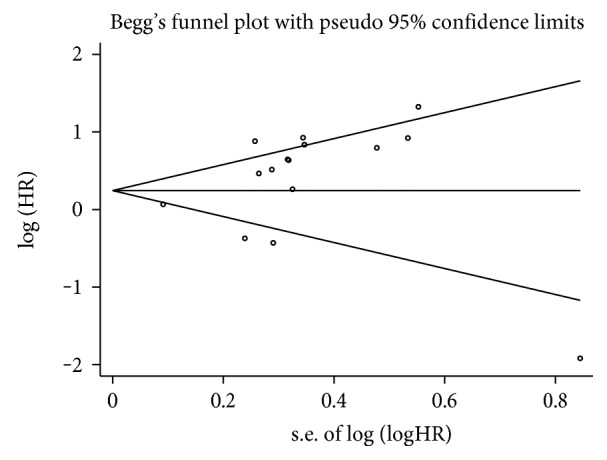
Begg's funnel plot for publication bias test of OS.

**Figure 6:**
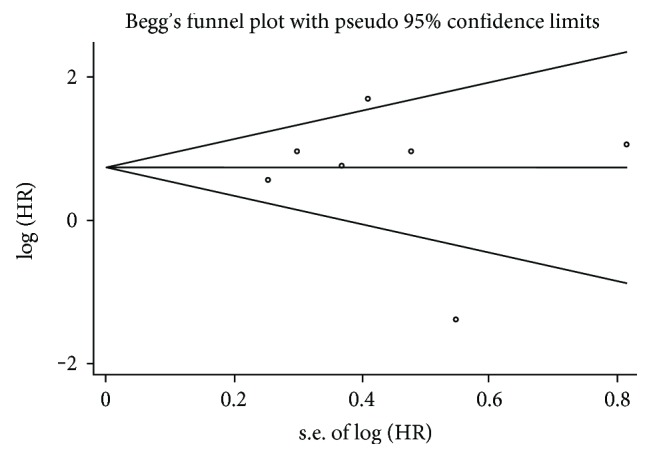
Begg's funnel plot for publication bias test of DFS/RFS/MFS.

**Figure 7:**
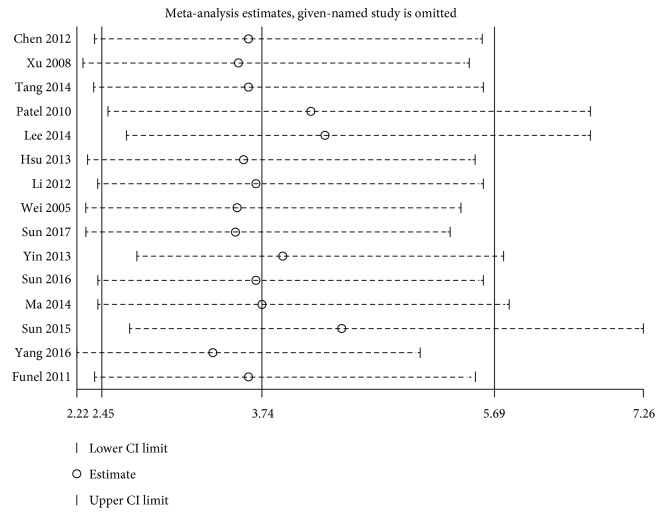
Sensitivity analysis on the relationship between loss of KLF4 expression and OS in digestive system cancers.

**Figure 8:**
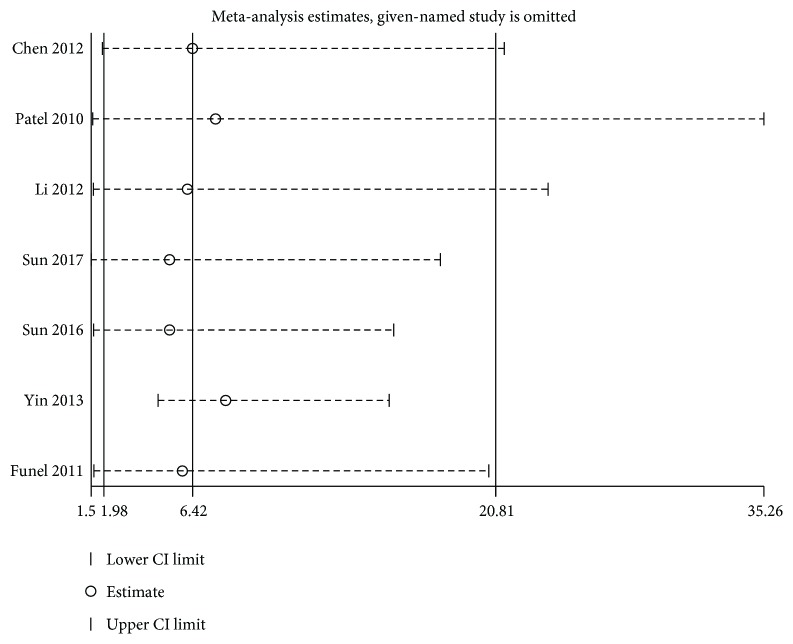
Sensitivity analysis on the relationship between loss of KLF4 expression and DFS/RFS/MFS in digestive system cancers.

The step of assessment for publication bias was fulfilled by qualitative Begg's funnel plot and the quantitative Egger's test. As shown in Figures 5 and 6, there was no obvious asymmetry. In addition, the Egger's test also indicated that there was no significant publication bias for OS (*P* = 0.155) and DFS/RFS/MFS (*P* = 0.761) in this meta-analysis. Meanwhile, the results of sensitivity analysis revealed robust stability of pooled HRs for the OS and DFS/RFS/MFS illustrated in Figure 7 and Figure 8, respectively. For the limited number of included studies (*n* = 2), both analyses were not performed for DSS.

## 4. Discussion

At least 16 distinct members constitute the Krüppel-like factor (KLF) family so far and are named for their similarity to Krüppel, a protein found in *Drosophila melanogaster* [5]. It is now well documented that, after binding to specific DNA sequences of target genes by their DNA-binding domain within carboxyl-terminal, KLFs play a pivotal role in regulating many important cellular functions such as cell proliferation, differentiation, growth, and apoptosis [36]. Among those factors, KLF4 is of full interest to researchers for its role as a tumor suppressor. KLF4 could inhibit tumor cell proliferation through inducing expression of p21 and/or p27 and downregulation of cyclinD1 [37]. During the epithelial-to-mesenchymal transition and metastatic process, KLF4 exhibits some suppressive effects for the ability of suppressing Snail and MMP-2 expression and promoting E-cadherin expression [38, 39]. Interestingly, mechanism studies indicate that micro-RNA molecules contribute to the negative expression of KLF4 proteins in some malignancies through binding to complementary sequences of KLF4. For example, oncogenic miRNAs such as miR-103 and miR-92a could promote cancer cell proliferation, invasion, and migration by inhibiting the expression of KLF4 [40, 41]. In addition, promoter hypermethylation and hemizygous deletion of KLF4 are also reported by researchers, leading to its expression suppression [17]. Recently, results from a phase 1 trial that have evaluated the effects of APTO-253, an inducer for KLF4, on patients with advanced solid tumors, finally showed its abilities against tumors and achievement for stable disease [42]. Furthermore, it has been reported that in several types of cancers, KLF4 may be a context-dependent oncogene, switching by a regulation on the expression levels of cell-cycle regulator p21 [43]. In the context of digestive system malignancies, a majority of investigations established potent evidence suggesting an unfavorable impact of loss of KLF4 expression on clinical prognosis. However, given that several literatures reported that KLF4 expression was a harmful prognostic indicator in some malignancies, it is very necessary to clarify the precise relation between KLF4 expression and prognostic value in patients diagnosed with digestive system cancers through a systematic review and meta-analysis, which may provide useful information for the application of targeted therapy on cellular KLF4 in the future.

To the best of our knowledge, this meta-analysis presented here is the first one to analyze the impact of loss of KLF4 expression on the survival of various digestive system malignancies. Briefly, a total of 17 studies including 2188 patients with distinct kinds of cancers yielded statistics, combined HRs, indicating significantly negative effect of loss of KLF4 expression on patients' survival time. Combined hazard ratios demonstrated that loss of KLF4 expression was associated with a poorer OS (HR = 1.61, 95% CI: 1.17–2.20, *P* = 0.003) and DFS/RFS/MFS (HR = 1.99, 95% CI: 1.12–3.52, *P* = 0.001) as well as DSS (HR = 1.73, 95% CI: 1.08–2.77, *P* = 0.022) in digestive system malignancies without regard to subgroup-confounding factors. In the subgroup analysis, most results of which were consistent with the corresponding overall result. But the existence of insignificant even opposing results should also be noted. First, in the subgroup analysis on the basis of ethnicity, loss of KLF4 expression yielded a better prognosis in Caucasian patients for DFS/RFS/MFS (HR = 0.59, 95% CI: 0.36–0.94) which is likely caused by the limited number of included studies (*n* = 2) or inherent differences of ethnicity. Second, as for different measurement methods, inconsistent results were obtained in patients detected by RT-PCR for OS and DFS/RFS/MFS (HR = 0.93, 95% CI: 0.62–1.39 and HR = 0.25, 95% CI: 0.09–0.73, resp.). The relatively small amounts of studies and the involvement of certain isoforms, in particular KLF*α* [44], may explain those discrepancies in some extent. Third, in the subgroup analysis according to the type of cancer, for ESCC patients, loss of KLF4 expression showed a trend of better prognosis though without statistical significance (HR = 0.83, 95% CI: 0.57–1.23, *P* = 0.355) which suggested that, similar to head and neck squamous cell carcinoma (HNSCC) [45], KLF4 might be a malignant transformation-related gene in ESCC, which still needs further investigation.

Based on the evidence presented in our meta-analysis, loss of KLF4 expression could be a poorer prognostic biomarker in most kinds of digestive system tumors except ESCC. Whereas, this study has several limitations. First, because of a limited amount of included studies of each type of cancers, the results of some carcinomas were statistically insignificant and might be less powerful. Second, the literatures were restricted to English-written papers, which probably introduced language bias. Third, the HRs of some literatures, extrapolated based on Tierney's method, were less reliable than those directly provided in the original articles. Fourth, the cut-off values in the studies were not uniform, which might be a source of heterogeneity. Fifth, significant heterogeneity existed in those studies recruited in this meta-analysis. Although we used a random-effect model and conducted subgroup analyses to explore the potential source of heterogeneity, there were unacceptable heterogeneities in those subgroups. Many factors could have contributed the heterogeneity observed among those studies, such as different population characteristics, pathological grade, histology type, or study designs. Finally, some publication bias was inevitable because positive results are more easily accepted by journals than negative or null results. Given all the above limitations, our results should be considered cautiously.

In conclusion, the present meta-analysis according to published articles demonstrated that loss of KLF4 expression was associated with poorer survival in most kinds of digestive system cancer patients, such as gastric, hepatic, pancreatic, and colorectal cancers. Additionally, although not statistically significant, we observed that loss of KLF4 expression predicted a trend of better survival in ESCC patients. At last, our results should be interpreted carefully for the aforementioned heterogeneity and limitations. To strengthen our findings, the prognostic value of KLF4 in digestive system malignancies should be further confirmed by large-scale and standard investigations, in particular, in those patients with ESCC.

## Figures and Tables

**Table 1:** Baseline characteristics of the seventeen included studies.

Author	Year	Region	Type	Stage	Number of patients	Follow-up (months)	Assay	Negative (*n*)	Cut off	Outcome	HR estimation	HR (95% CI)	NOS score
Chen	2012	China	CRC	I–IV	99	NA	IHC	34	Low expression	OS	SC	2.62 (1.88–7.18)	6
							IHC	34	Low expression	MFS	SC	2.88 (1.09–26.54)	
Xu	2008	China	CRC	I–IV	60	NA	IHC	42	Negative expression	OS	SC	1.81 (0.91–2.77)	6
Tang	2014	China	CRC	I–IV	85	NA	RT-PCR	42	Low expression	OS	SC	2.08 (1.54–5.26)	7
Patel	2010	USA	CRC	I–IV	365	NA	IHC	249	<10% staining	OS	SC	1.08 (1.03–1.47)	7
							IHC	249	<10% staining	DFS	SC	1.75 (1.06–2.86)	
Lee	2014	South Korea	CRC	I–IV	125	0.4–96.3	RT-PCR	80	<2150 copies/*μ*l	OS	SC	0.61 (0.44–1.35)	8
Hsu	2013	China	GC	I–IV	118	NA	IHC	31	Low expression	OS	SC	1.71 (1.03–2.85)	8
Li	2012	China	GC	I–IV	264	9–69	IHC	150	IRS ≤ 1	OS	Reported	2.89 (1.18–9.23)	8
							IHC	150	IRS ≤ 1	DFS	Reported	2.14 (1.03–4.37)	
Wei	2005	USA	GC	I–IV	39	NA	IHC	27	IRS ≤ 3	OS	SC	2.10 (1.14–3.87)	7
Sun	2017	China	HCC	I–III	148	NA	IHC	67	IRS ≤ 3	OS	Reported	2.91 (1.50–5.66)	8
							IHC	67	IRS ≤ 3	RFS	Reported	2.60 (1.45–4.68)	
Hsu	2014	China	HCC	I–IV	205	2.4–147.6	IHC	160	Staining intensity ≤ 1+	DSS	SC	2.51 (1.18–5.16)	8
Sun	2016	China	HCC	I–III	98	NA	IHC	29	Negative expression	OS	Reported	4.59 (1.59–13.34)	8
							IHC	29	Negative expression	RFS	Reported	5.42 (2.42–12.06)	
Yin	2013	China	HCC	I–III	57	5–58	RT-PCR	50	Low expression	OS	SC	0.11 (0.02–0.52)	8
							RT-PCR	50	Low expression	RFS	SC	0.25 (0.09–0.77)	
Shimada	2012	Japan	ESCC	I–IV	80	40	IHC	50	IRS ≤ 3	DSS	SC	1.34 (0.73–2.47)	8
Ma	2014	China	ESCC	I–III	98	3–72	IHC	55	IRS ≤ 3	OS	SC	1.35 (0.72–2.53)	8
Sun	2015	China	ESCC	I–IV	149	NA	IHC	95	IRS ≤ 4	OS	SC	0.65 (0.41–1.03)	7
Yang	2016	China	PDAC	I–IV	106	24	IHC	59	<25% staining	OS	SC	2.76 (1.68–4.52)	8
Funel	2011	Italy	PDAC	NA	22	11.6–55.2	IHC	16	Negative expression	OS	Reported	2.50 (1.00–6.30)	7
							IHC	16	Negative expression	DFS	Reported	2.60 (1.00–6.50)	

CRC: colorectal cancer; GC: gastric cancer; HCC: hepatocellular carcinoma; ESCC: esophageal squamous cell carcinoma; PDAC: pancreatic ductal adenocarcinoma; IHC: immunohistochemistry; RT-PCR: reverse transcription polymerase chain reaction; IRS: immunoreaction score; OS: overall survival; DFS: disease-free survival; DSS: disease-specific survival; MFS: metastasis-free survival; DSS: disease-specific survival; RFS: recurrence-free survival; SC: survival curve; NA: not available; 95% CI: 95% confidence interval; HR: hazard ratio; NOS: Newcastle-Ottawa scale.

**Table 2:** Subgroup analysis of loss of KLF4 expression for OS and DFS/RFS/MFS in digestive system cancers.

Outcome	Variables	Number of studies	Model	HR (95% CI)	Heterogeneity	
					*I* ^2^ (%)	*p* value
OS		15	Random	1.61 (1.17–2.20)	78.20%	0.001
	*Cancer type*					
	CRC	5	Random	1.17 (1.01–1.37)	76.80%	0.002
	GC	3	Fixed	1.97 (1.36–2.83)	0.00%	0.646
	HCC	3	Random	2.30 (1.35–3.92)	87.10%	0.001
	ESCC	2	Random	0.84 (0.58–1.22)	70.40%	0.066
	PDAC	2	Fixed	2.70 (1.75–4.17)	0.00%	0.853
	*Ethnicity*					
	Caucasian	3	Random	1.17 (1.00–1.38)	71.10%	0.032
	Asian	12	Random	1.54 (1.28–1.84)	79.00%	0.001
	*Method*					
	IHC	12	Random	1.38 (1.21–1.57)	75.80%	0.001
	RT-PCR	3	Random	0.93 (0.62–1.39)	87.00%	0.001
DFS/MFS/RFS		7	Random	1.99 (1.12–3.52)	72.50%	0.001
	*Cancer type*					
	CRC	2	Fixed	1.83 (1.14–2.94)	0.00%	0.559
	HCC	3	Random	2.20 (1.43–3.39)	90.40%	0.001
	GC	1	—	2.14 (1.04–4.41)	—	—
	PDAC	1	—	2.60 (1.02–6.63)	—	—
	*Ethnicity*					
	Caucasian	2	Random	0.59 (0.36–0.94)	72.50%	0.001
	Asian	5	Random	1.91 (1.23–2.96)	81.00%	0.001
	*Method*					
	IHC	6	Fixed	2.43 (1.82–3.25)	13.00%	0.332
	RT-PCR	1	—	0.25 (0.09–0.73)	—	—

CRC: colorectal cancer; GC: gastric cancer; HCC: hepatocellular carcinoma; ESCC: esophageal squamous cell carcinoma; PDAC: pancreatic ductal adenocarcinoma; IHC: immunohistochemistry; RT-PCR: real-time polymerase chain reaction.
